# *Salmonella* Typhimurium effector SseI inhibits chemotaxis and increases host cell survival by deamidation of heterotrimeric G_i_ proteins

**DOI:** 10.1371/journal.ppat.1007248

**Published:** 2018-08-13

**Authors:** Thorsten Brink, Veronika Leiss, Peter Siegert, Doris Jehle, Julia K. Ebner, Carsten Schwan, Aliaksei Shymanets, Sebastian Wiese, Bernd Nürnberg, Michael Hensel, Klaus Aktories, Joachim H. C. Orth

**Affiliations:** 1 Institut für Experimentelle und Klinische Pharmakologie und Toxikologie, Medizinische Fakultät, Albert-Ludwigs-Universität Freiburg, Freiburg, Germany; 2 Abteilung für Pharmakologie und Experimentelle Therapie, Medizinische Fakultät und ICePhA, Eberhard-Karls-Universität Tübingen, Germany; 3 Spemann Graduate School of Biology and Medicine (SGBM), Albert-Ludwigs-Universität Freiburg, Freiburg, Germany; 4 Fakultät für Biologie, Albert-Ludwigs-Universität Freiburg, Freiburg, Germany; 5 Zentrum für Biosystemanalyse, Albert-Ludwigs-Universität Freiburg, Freiburg, Germany; 6 Abteilung Mikrobiologie, Fachbereich Biologie/Chemie, Universität Osnabrück, Osnabrück, Germany; 7 BIOSS Centre for Biological Signalling Studies, Albert-Ludwigs-Universität Freiburg, Freiburg, Germany; University of California, Davis, UNITED STATES

## Abstract

*Salmonella enterica* serotype Typhimurium (*S*. Typhimurium) is one of the most frequent causes of food-borne illness in humans and usually associated with acute self-limiting gastroenteritis. However, in immunocompromised patients, the pathogen can disseminate and lead to severe systemic diseases. *S*. Typhimurium are facultative intracellular bacteria. For uptake and intracellular life, *Salmonella* translocate numerous effector proteins into host cells using two type-III secretion systems (T3SS), which are encoded within *Salmonella* pathogenicity islands 1 (SPI-1) and 2 (SPI-2). While SPI-1 effectors mainly promote initial invasion, SPI-2 effectors control intracellular survival and proliferation. Here, we elucidate the mode of action of *Salmonella* SPI-2 effector SseI, which is involved in control of systemic dissemination of *S*. Typhimurium. SseI deamidates a specific glutamine residue of heterotrimeric G proteins of the Gα_i_ family, resulting in persistent activation of the G protein. G_i_ activation inhibits cAMP production and stimulates PI3-kinase γ by Gα_i_-released Gβγ subunits, resulting in activation of survival pathways by phosphorylation of Akt and mTOR. Moreover, SseI-induced deamidation leads to non-polarized activation of Gα_i_ and, thereby, to loss of directed migration of dendritic cells.

## Author summary

*Salmonella* Typhimurium is one of the most common causes of gastroenteritis in humans. In immunocompromised patients, the pathogen can cause systemic infections. Crucial virulence factors are encoded on two *Salmonella* pathogenicity islands SPI-1 and SPI-2. While SPI-1 encodes virulence factors essential for host cell invasion, intracellular proliferation of the pathogen depends mainly on SPI-2 effectors. Here, we elucidate the mode of action of *Salmonella* SPI-2 effector SseI. SseI activates heterotrimeric G proteins of the Gα_i_ family by deamidation of a specific glutamine residue. Deamidation blocks GTP hydrolysis by Gα_i_, resulting in a persistently active G protein. G_i_ activation inhibits cAMP production and stimulates PI3Kγ by Gα_i_-released Gβγ subunits, resulting in activation of survival pathways by phosphorylation of Akt and mTOR. Moreover, deamidation of Gα_i_ leads to a loss of directed migration in dendritic cells. The data offers a new perspective in the understanding of the actions of SseI.

## Introduction

*Salmonella enterica* serovars are pathogenic bacteria that cause severe diseases ranging from enteric fever (e.g. by *Salmonella* Typhi) to gastroenteritis and bacteraemia caused by non-typhoidal *Salmonella* (NTS). *Salmonella* Typhimurium, the model organism of NTS infection, has a broad host spectrum and is one of the most frequent causes of food-borne illness in humans and other vertebrates including food-producing animals. *S*. Typhimurium infection is usually associated with acute self-limiting gastroenteritis in immunocompetent individuals. However, in immunocompromised patients, *S*. Typhimurium can disseminate and lead to severe systemic diseases [1–4].

*S*. Typhimurium are facultative intracellular bacteria, which exploit uptake by phagocytic intestinal cells but are also able to force their uptake into non-phagocytic epithelial cells [5]. Inside host cells, *Salmonella* reside and proliferate in a specific membrane compartment defined as *Salmonella*-containing vacuole (SCV). Uptake and intracellular life of *Salmonella* depends on two type-III secretion systems (T3SS) that are encoded within *Salmonella* pathogenicity islands 1 (SPI-1) and 2 (SPI-2). These T3SSs act as molecular syringes that translocate > 40 *Salmonella* effector proteins into the host cell cytosol. While initial invasion is mainly promoted by SPI-1 T3SS, intracellular survival and proliferation largely depends on SPI-2 T3SS effectors [6–9].

At least 28 effectors are secreted by the SPI-2 T3SS into host cells. A “core” subset of effectors (e.g., SseF, SseG, SifA, and PipB2) appear to be involved in organization and maturation of *Salmonella* containing vacuoles (SCV) [9]. Other effectors play major roles in suppression of innate immune signaling pathways or modulate adaptive immune responses [9–12]. Recently, the SPI-2 effector SseI (also known as SrfH) has attracted increased attention, because it inhibits directed migration of dendritic cells and is involved in long-term systemic infection [13]. Moreover, pseudogenization of the effector gene *sseI* confers rapid systemic hyperdissemination of *S*. Typhimurium (sequence type) ST313, which commonly causes systemic bacteremia in children and immunocompromised adults in sub-Saharan Africa [14]. SseI consists of 322 amino acids and its N-terminal part is similar to other SPI-2 effectors, suggesting a role in translocation and membrane localization. In fact, cysteine-9 of SseI has been shown to be palmitoylated in host cells to achieve membrane binding [15]. Until now, however, the molecular mechanism of SseI has remained unknown. Because crystallographic studies suggested that the 37 kDa SseI effector protein exhibits structural similarity with the catalytic domain of the deamidating *Pasteurella multocida* toxin (PMT) [16], we studied whether SseI possesses deamidase activity.

Deamidation is a post-translational modification, which is exploited by various bacterial exotoxins and effectors [17, 18]. A prototype of these exotoxins is PMT [17, 19, 20]. This exotoxin is a 145 kDa protein that is responsible for atrophic rhinitis in pigs. The toxin activates osteoclast differentiation, while differentiation of osteoblasts is blocked [21, 22]. The underlying molecular mechanism of the action of PMT is the activation of heterotrimeric G proteins by deamidation [23]. PMT deamidates a specific glutamine residue in the α-subunits of G_q/11_, G_i/o_ and G_12/13_ proteins, which plays a crucial role in hydrolysis of GTP and in inactivation of heterotrimeric G proteins [24]. Thus, deamidation of the glutamine residue by PMT freezes the G protein in its active state.

Here, we elucidated the molecular mode of action of SseI. We report that the *Salmonella* SPI-2 T3SS effector deamidates heterotrimeric G proteins of the G_i_ family *in vitro* and *in vivo*. Thereby, SseI is responsible for the activation of Akt kinase after target cell invasion and increases cell survival during *Salmonella* infection. Moreover, our studies reveal a pivotal role of SseI-induced deamidation of Gα_i_ in the inhibition of dendritic cell migration.

## Results

### Characterization of the deamidation of G proteins by SseI *in vitro*

**Fig 1:**
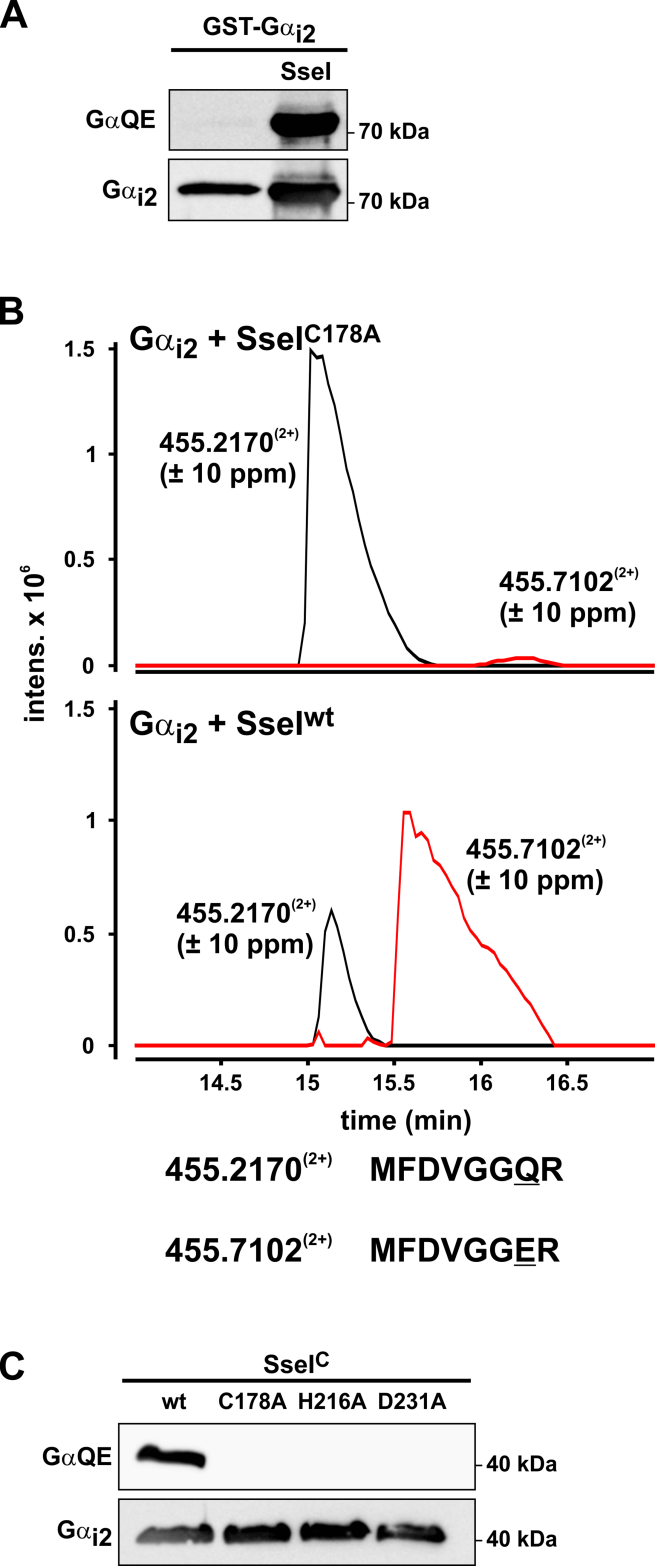
SseI deamidates an essential glutamine residue in the switch II region of Gα_i2_. **(A)** Western blot analysis of Gα_i2_. GST-Gα_i2_ was coexpressed without (control) and with SseI in *E*. *coli*. Purified GST-Gα_i2_ was immunoblotted and detected by the Gα_i2_-specific antibody (Gα_i2_) and by the deamidation-specific antibody (GαQE). **(B)** Gα_i2_ coexpressed with SseI was analyzed by HPLC-MS/MS spectrometry. Combined extracted ion chromatograms for m/z 455.2^(2+)^ and 455.7^(2+)^, corresponding to the tryptic peptides MFDVGGQR and MFDVGGER (amino acids 199–206) of Gα_i2_, are shown (see also S1B and S1C Fig). (Upper panel) Gα_i2_ coexpressed with inactive SseI-C178A. (Lower panel) Gα_i2_ coexpressed with active wt SseI. **(C)** Immunoblot analysis of recombinantly expressed Gα_i2_ incubated with wild type C-terminal part of SseI^C^ (wt) or with 3 different mutant SseI^C^ (C178A, H216A and D231A).

The comparison of the amino acid sequence of the deamidase domain of PMT (residues 1144–1240) with SseI revealed high sequence similarity (S1A Fig). More importantly, amino acids known to be crucial for deamidase activity of PMT are conserved in SseI (e.g., C178, H216, and D231) [19]. Therefore, we utilized a previously described method to detect deamidation of G proteins by bacterial effectors [23, 24]. To this end, SseI was coexpressed in *E*. *coli* with the α-subunit of G_i2_. Deamidation of Gα_i2_ was determined by immunoblot analysis, utilizing a monoclonal antibody (GαQE) that specifically recognizes Gα after deamidation of a specific glutamine residue in the switch II region [25]. The GαQE antibody detected Gα_i2_ coexpressed with SseI in *E*. *coli* but not the solely expressed Gα_i2_, indicating a deamidation activity of SseI (Fig 1A). Purified Gα_i2_ was subjected to mass spectrometric (MS) analysis. MS analysis identified a tryptic peptide of Gα_i2_ (m/z 455.2170^2+^) corresponding to amino acid residues 199–206. This peptide includes the glutamine residue (Gln-205) essential for the hydrolysis of GTP. Additionally, a second peptide was identified with a mass shift of 1 Da (m/z 455.7102^2+^). Tandem MS analysis revealed that Gln-205 was deamidated, resulting in a glutamic acid residue at this position. No relevant deamidation occurred when Gα_i2_ was coexpressed with mutant SseI (SseI-C178A), which lacks deamidation activity (Fig 1B and S1B and S1C Fig). Similarly, recombinantly expressed wild type (wt) SseI (amino acids 137–322, SseI^C^), but not the C178A, H216A, or D231A mutants, caused deamidation of purified Gα_i2_ (Fig 1C).

### Construction of a PMT-SseI chimeric toxin

**Fig 2:**
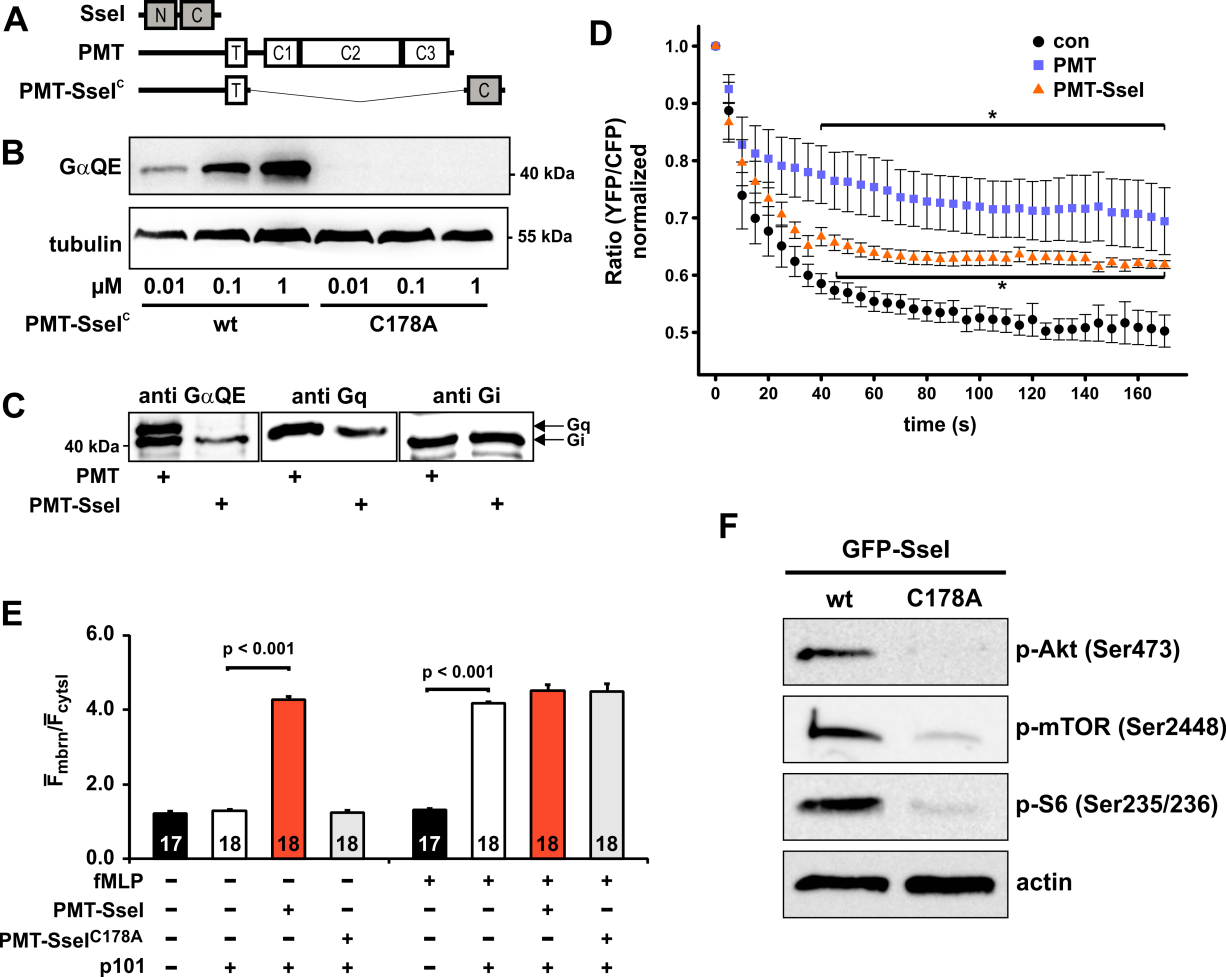
Cell permeable SseI activates Gα_i_-dependent signal transduction pathways. **(A)** Schematic representation of the cell permeable PMT-SseI^C^ chimera. The C-terminal domain of SseI, encompassing amino acids 137–322 (SseI^C^), was fused to the receptor binding and translocation domain of PMT (PMT, amino acids 1–505). **(B)** Immunoblot analyses of HEK-293 cells incubated with indicated concentrations of PMT-SseI^C^ or PMT-SseI^C^-C178A for 16 h. RIPA buffer lysates were prepared and immunoblots were performed to detect Gα deamidation, using the GαQE antibody. Equal loading was verified by detection of tubulin. **(C)** Comparison of PMT- and PMT-SseI^C^-induced Gα deamidation. PMT treatment of cells (1 nM, 16 h) led to two signals of deamidated Gα proteins in immunoblot analysis, migrating at the same size of Gα_i_ and Gα_q_. PMT-SseI^C^ (100 nM, 16 h) induced one deamidation signal at the size of Gα_i_. **(D)** PMT-SseI^C^ blocks stimulation of the adenylyl cyclase (AC) activity. HeLa cells were transfected overnight (16 h) with the FRET sensor construct EPAC2-camps. Cells were left untreated (con) or were incubated with PMT (1 nM) or PMT-SseI^C^ (100 nM) for 4 h. AC was stimulated with forskolin (40 μM, added at time point 0) and FRET measurement was performed. cAMP increase is depicted as normalized ratio of YFP/CFP of the sensor. **(E)** PMT-SseI^C^ stimulates the PI3Kγ. HEK-293 cells were transfected with the PIP_3_ sensor GFP-Grp1_PH_ and the PI3-kinase subunit p110γ. In addition, HEK-293 cells were transfected with the non-catalytic PI3-kinase γ subunit p101 as indicated. Cells were stimulated with PMT-SseI^C^ or the inactive C178A mutant of PMT-SseI^C^ (each 100 nM). After baseline measurement for 1 min fMLP (1 μM) was added and the measurement was continued for 5 min. Histogram shows the quantification of the membrane translocation of GFP-Grp1_PH_. Data depicted represent the mean ±SEM from 3 independent sets of experiments analyzing 17 or 18 cells in total. **(F)** Immunoblot analysis of BMDMs transiently transfected with GFP-SseI or GFP-SseI-C178A. Cells were incubated for 24 h, followed by serum starvation for 4 h. Cells were lysed and subjected to Western blot analysis with indicated antibodies. Representative immunoblots from one experiment are shown. See also S2 Fig.

The effector SseI is secreted by a type III secretion system of *S*. Typhimurium. Therefore, SseI is not taken up by eukaryotic cells as compared to AB-type bacterial exotoxins like PMT. To enable cellular uptake of SseI, we utilized the receptor binding and translocation domain of PMT [26]. The deamidation domain of SseI (SseI^C^) was fused C-terminally to the N-terminal part of PMT (amino acids 1–505) (Fig 2A). This chimera, PMT-SseI^C^, was recombinantly expressed, purified and tested for cellular activity. Treatment of HEK-293 cells with increasing concentrations of PMT-SseI^C^ led to a deamidation of Gα proteins as determined by immunoblot analysis using the GαQE antibody. Treatment of cells with the C178A mutant of PMT-SseI^C^ exhibited no deamidation (Fig 2B). In a next step, we compared the activity of PMT-SseI^C^ with that of PMT. HEK-293 cells were intoxicated either with PMT or with PMT-SseI^C^ and cell lysates were tested for deamidated Gα proteins. PMT led to two deamidation signals migrating at the molecular mass of the Gα_q_ and Gα_i_ proteins (Fig 2C). However, PMT-SseI^C^ only induced one deamidation signal migrating at the molecular mass of Gα_i_.

### Activation of G_i_ proteins by the chimeric toxin PMT-SseI^C^

Deamidation of the essential glutamine residue in the switch II region impairs the GTP hydrolysis by the α-subunit resulting in a permanent active phenotype of the G protein [23]. Activated Gα_i_ family members inhibit adenylyl cyclase leading to decreased cAMP levels. We utilized a FRET-based approach to determine how cAMP levels respond to SseI treatment. The FRET sensor Epac2-camps [27] exhibits a decreased FRET ratio, when cAMP is increased. When HeLa cells, expressing the FRET sensor, were treated with the adenylyl cyclase activator forskolin, the FRET ratio declined, indicating increased cAMP levels. In line with an activation of Gα_i_, pretreatment with PMT-SseI^C^ or PMT attenuated the effect of forskolin, indicating an inhibitory effect on adenylyl cyclase (Fig 2D). Similar results were obtained by direct measurements of cAMP levels (see ELISA assay S2A Fig).

Activation of heterotrimeric G proteins leads to dissociation into Gα and Gβγ-subunits [28]. Both subunits can interact with their specific effectors and stimulate specific signaling cascades. Therefore, we studied the impact of SseI on phosphoinositol-3-kinase (PI3K)γ, a prototypical Gβγ effector [29–31]. Activation of Gβγ was monitored by translocation of the phosphatidylinositol-3, 4, 5-triphosphate (PIP_3_) sensor protein GFP-Grp1_PH_ from the cytosol to the membrane after additional ectopic expression of p110γ without and with p101 (Fig 2E and S2B Fig). As a control, we used N-formylmethionine-leucyl-phenylalanine (fMLP), which stimulates Gi-coupled GPCRs (G protein-coupled receptors). fMLP induced translocation of Grp1_PH_ to the membrane in the presence of complete PI3Kγ (p101 with p110γ) (S2Bb Fig). However, in the absence of complete PI3Kγ (p101 without p110γ), fMLP did not redistribute Grp1_PH_, indicating the insensitivity of endogenously expressed PI3Ks (S2Ba Fig). Treatment of transfected cells with PMT-SseI^C^, but not with the inactive mutant, strongly stimulated the redistribution of Grp1_PH_ to the plasma membrane (Fig 2E and S2Bc/d Fig). This effect was not further stimulated by fMLP incubation, indicating an activation of the PI3K independent of the stimulation by GPCRs.

### Activation of Akt signaling by SseI

Various recent studies suggested that Gα_i/o_ proteins and PI3-kinases play crucial roles in regulation of immune cell signaling and cell survival in infection [32–36]. Therefore, we studied the downstream signaling of Gβγ subunits and of PI3Kγ in more detail. To this end, we transfected bone marrow-derived macrophages (BMDMs) or RAW264.7 macrophages with a GFP expression vector either harboring SseI or the catalytically inactive mutant (SseI-C178A). After starvation of cells for 4 h, phosphorylation of Akt, mTOR and the mTOR-effector S6 ribosomal protein was analyzed by immunoblotting. Transfection with SseI strongly increased phosphorylation of the PI3K downstream effectors Akt, mTOR, and S6 ribosomal protein in BMDMs (Fig 2F and S2C Fig). Similar results were obtained in RAW264.7 macrophages (S2D and S2E Fig).

### Role of SseI in infection of macrophages by *Salmonella* Typhimurium

**Fig 3:**
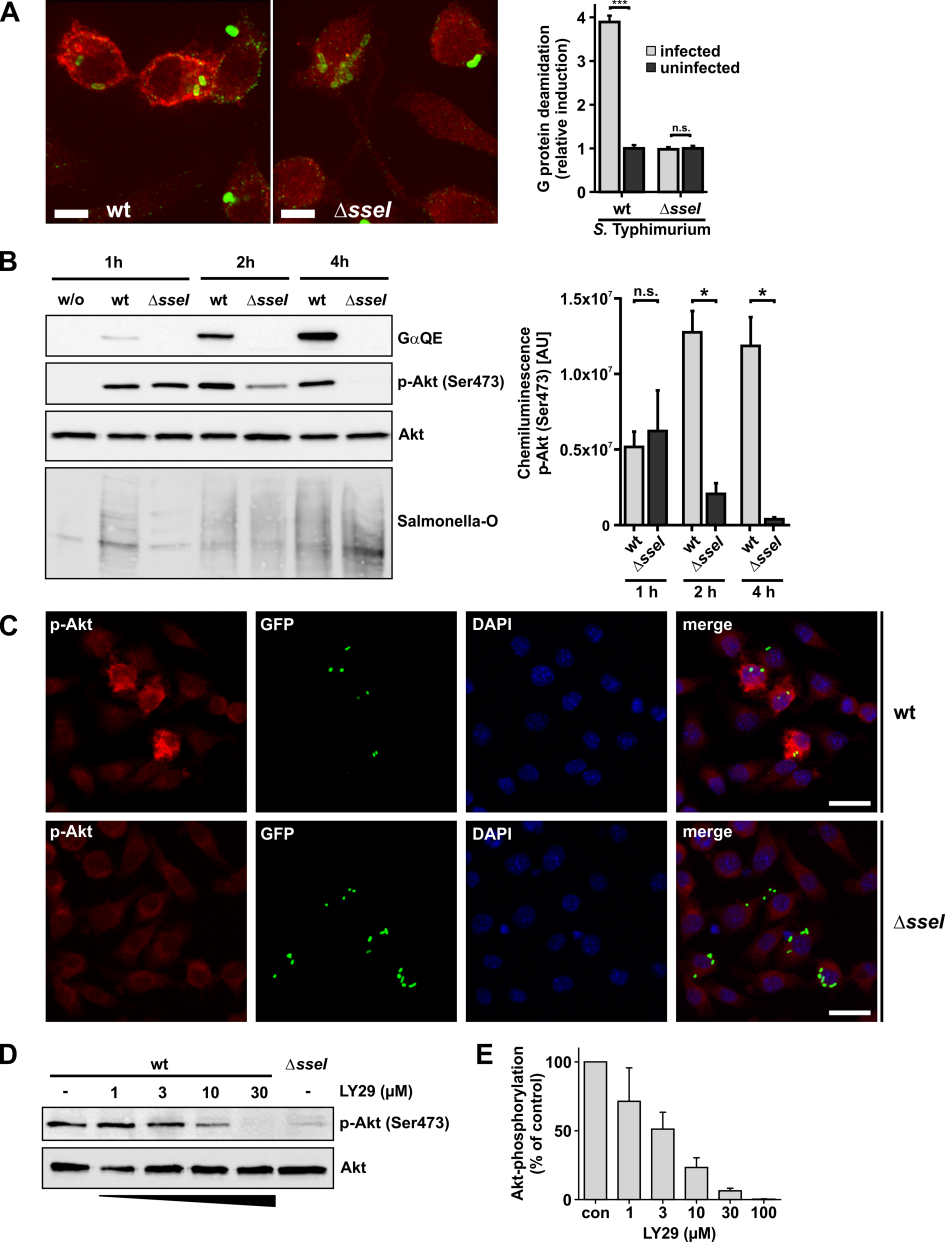
Cellular effects of SseI during infection. **(A)** Fluorescence microscopy of fixed cells. RAW264.7 cells were infected with wild type (wt) *S*. Typhimurium or a Δ*sseI*-strain at a MOI of 1 for 5 h. Deamidation of Gα was detected utilizing the GαQE antibody and an Alexa 568-conjugated secondary antibody. *Salmonella* were identified by *Salmonella* O antiserum and an Alexa 488-conjugated secondary antibody. Deamidated Gα is depicted in red and *Salmonella* in green. Z planes showing Salmonella were maximum projected into one image. Scale bars = 5 μm. Quantification of images (right panel). Gα protein deamidation of *Salmonella*-infected or uninfected cells was calculated by determining the average intensity of Alexa 568 fluorescence of the whole cell area in the Z plane with internalized *Salmonella*. Significance was determined by two-tailed Student’s *t*-test. Data are means ±SEM. (n = 10 cells). **(B-D)** Serum-starved RAW264.7 cells were infected with a MOI of 30 for 30 min. **(B)** Time-resolved immunoblot analysis. At indicated times p.i., cells were lysed and processed for immunoblotting. Blotting membranes were treated with indicated antibodies (GαQE, p-Akt and Akt). Representative blots of 3 independent experiments are shown. Equal loading was verified by detection of Akt, presence of *Salmonella* was verified by *Salmonella*-O antigen staining. Right panel shows the quantification of p-Akt (Ser473) labeling over time after infection with wt- and Δ*sseI*-*Salmonella* strains from 3 independent experiments. Chemiluminescence intensity, given as area units [AU], was determined for each band. Statistical significance was assessed by Mann Whitney test. **(C)** Only macrophages infected with wild type (wt) *Salmonella* show strong phosphorylation of Akt. Cells were infected with pEGFP expressing wt- or *ΔsseI-Salmonella* Typhimurium for 30 min. 5 h p.i. cells were fixed and processed for immunofluorescence microscopy. Cells were stained for phospho-Akt Ser473 (red) and nuclei were stained with DAPI (blue). Scale bars = 20 μm. **(D)** Addition of the PI3K inhibitor LY29 leads to concentration dependent inhibition of Akt phosphorylation in wild type (wt) *Salmonella-*infected macrophages. 1.5 h p.i. cells were treated with the indicated concentrations of LY29 for 3.5 h. Thereafter, cells were lysed. Lysates were processed for immunoblotting with indicated antibodies (Akt, p-Akt(Ser473)). **(E)** Quantification of p-Akt (Ser473) immunoblots of infected cells treated with increased concentrations of LY29. Experiments were performed as depicted in (D). Intensity of labeled bands was normalized to untreated (con) cells. Values are means ±SEM from at least 3 independent experiments.

To study the role of SseI in infection of RAW264.7 macrophages and BMDMs, we employed wt- or Δ*sseI*-*Salmonella* Typhimurium strains and used immunofluorescence microscopy. As depicted in Fig 3A, *S*. Typhimurium were identified by *Salmonella* O-antigen antiserum inside and outside of macrophages. Notably, we observed deamidated G proteins, determined by the GαQE antibody, only in macrophages infected with wt-*Salmonella*. Moreover, deamidation occurred strictly after internalization of bacteria (Fig 3A, S3A and S3B Fig).

Next, we studied the activation of the PI3K/Akt pathway by SseI during infection of RAW264.7 macrophages with wt- or Δ*sseI*-*Salmonella* strains. Immunoblot analysis revealed G protein deamidation as early as ~2 h post infection (p.i.) with subsequent increase over time (Fig 3B). Infection with wt- and Δ*sseI-Salmonella* strains led to phosphorylation of Akt at 1 h p.i.. Hereafter, phosphorylation of Akt decreased over time in macrophages infected with Δ*sseI-Salmonella*. In wt-*Salmonella*-infected macrophages, phosphorylation of Akt remained longer than in the Δ*sseI-Salmonella-*infected cells (Fig 3B). Immunofluorescence microscopic studies revealed that 5 h p.i. an increase in Akt-phosphorylation was seen in wt-*Salmonella*-infected cells, but not in non-infected or Δ*sseI-*infected cells (Fig 3C and S3C Fig). In line with an essential role of PI3K, LY294002 (LY29), a specific PI3K inhibitor, decreased phosphorylation of Akt in a concentration-dependent manner (Fig 3D and 3E).

**Fig 4:**
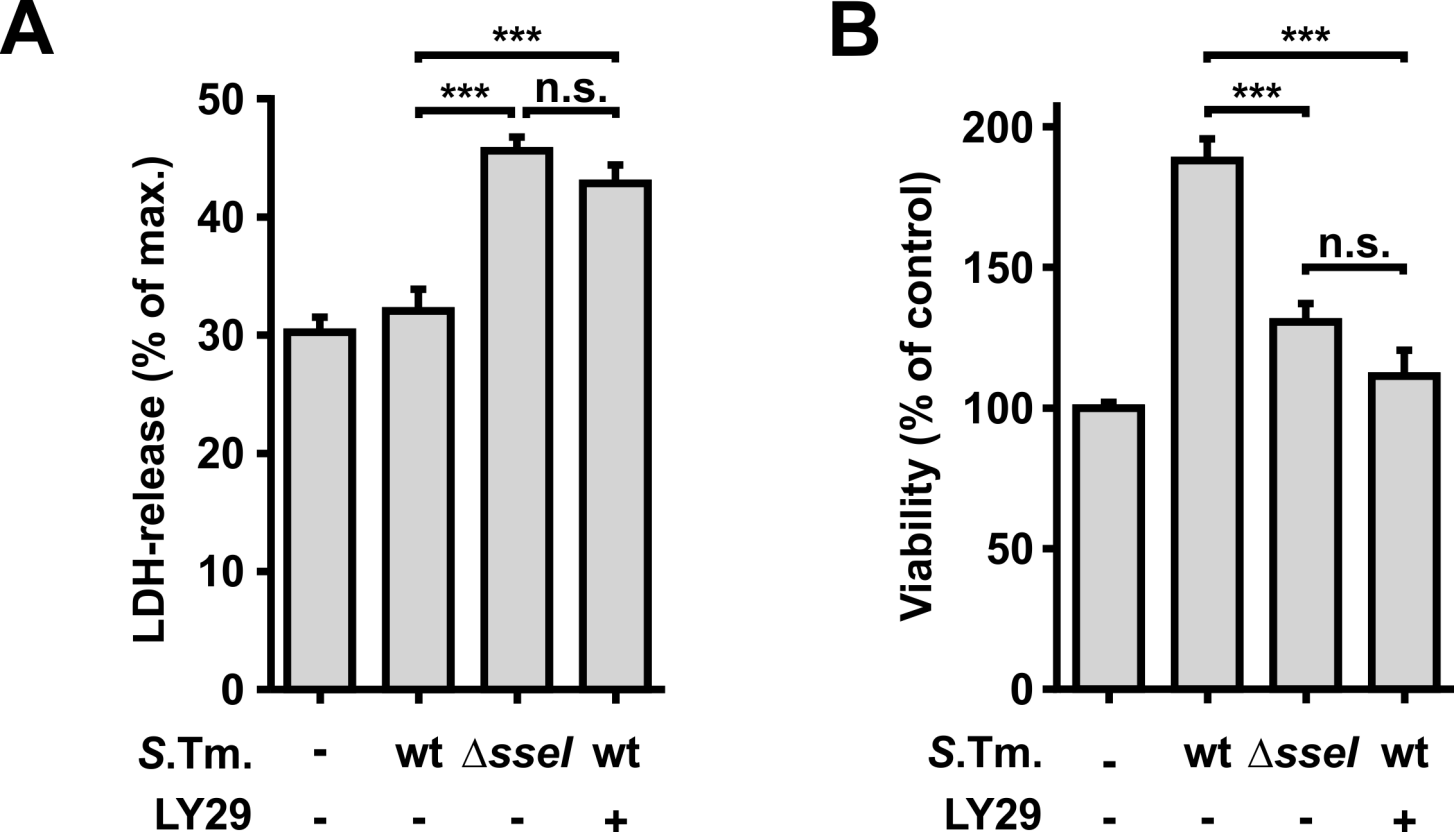
Effects of SseI on host cell survival. **(A and B)** RAW264.7 macrophages were infected with wild type (wt) or Δ*sseI*-*Salmonella* (MOI = 30; 30 min). 1.5 h p.i., cells were starved for 3.5 h in Earle’s balanced salt solution. The PI3K inhibitor LY29 (30 μM) was added 1.5 h p.i. where indicated. **(A)** The amount of LDH released into the medium was measured fluorometrically 5 h p.i.. Δ*sseI-*infected macrophages showed higher amounts of released LDH compared to wt-*Salmonella* infected cells. Data show means ±SEM from 3 independent experiments. Maximum LDH-release was obtained in the presence of 1% Triton X-100. Statistical significance was assessed using ANOVA (Bonferroni post-test). **(B)** Viability of RAW264.7 macrophages was fluorometrically measured 5 h p.i. by the ability of cells to reduce Resorufin. Experiments were performed in triplicates. Values are means ±SEM from at least 3 independent experiments. Statistical significance was assessed using ANOVA (Bonferroni post-test).

Several publications have shown that activation of Akt leads to anti-apoptotic and pro-survival outcomes in various mammalian cell types [33, 36]. Therefore, we were prompted to investigate, whether the infection with wt- or Δ*sseI-Salmonella* has an effect on the survival of host cells. To this end, we measured cell death of infected cells by determination of LDH release from infected RAW264.7 macrophages. This study revealed that infection with Δ*sseI*-*Salmonella* caused higher amounts of LDH release than with wt-*Salmonella* (Fig 4A). Accordingly, cell viability measured by the metabolic capacity of the cells, was increased after infection with wt-*Salmonella* as compared to Δ*sseI*-infected cells (Fig 4B). Inhibition of PI3K with LY29 diminished the effects of wt-*Salmonella* (Fig 4A and 4B).

### Effect of SseI on migration of dendritic cells

**Fig 5:**
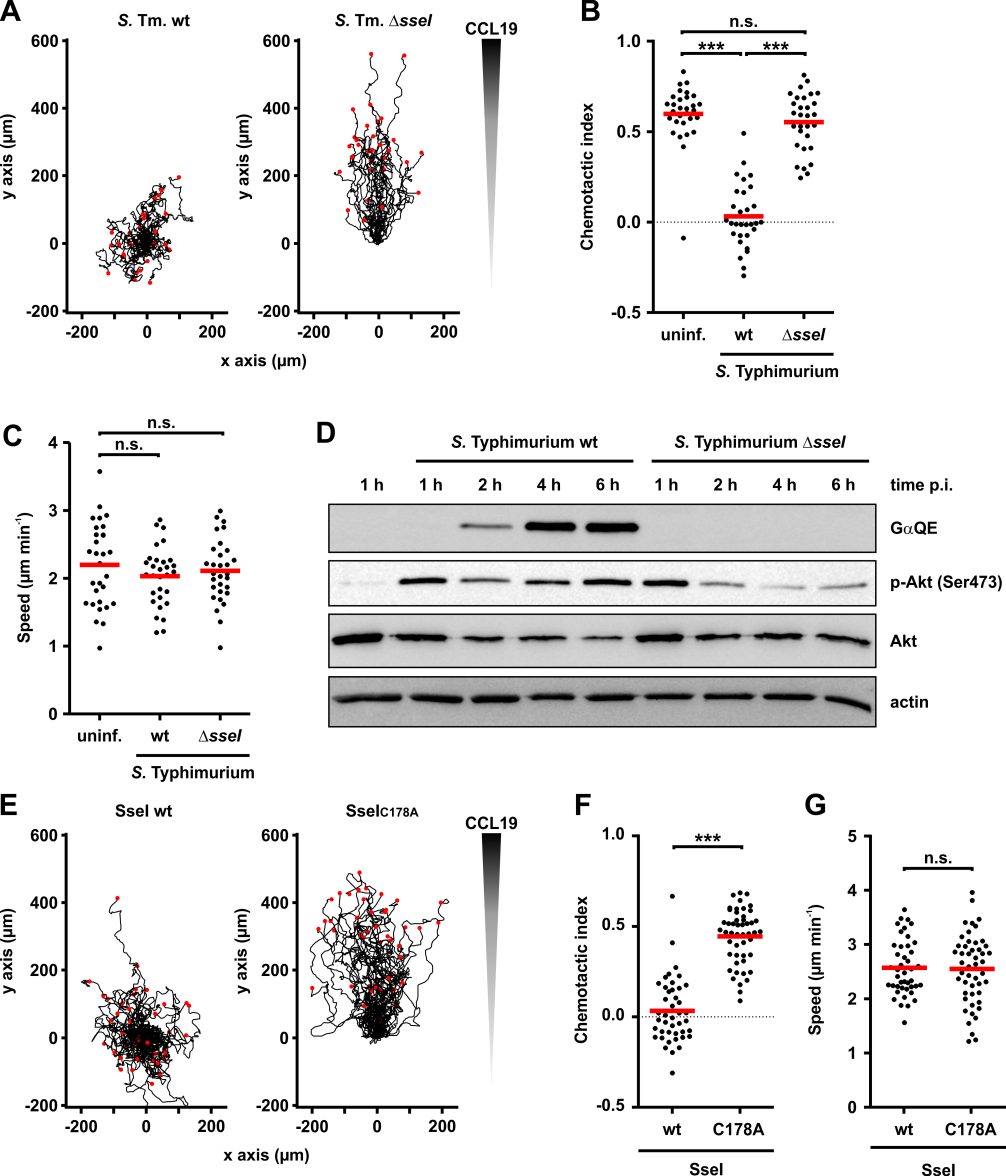
Influence of SseI on directed migration of dendritic cells. **(A-C)** DCs were infected with GFP-expressing wild type (wt)- or Δ*sseI-Salmonella* (MOI = 30; 30 min). Then, cells were transferred into a collagen matrix. **(A)** A CCL19 gradient was applied and directed migration for 4 h was determined via time-lapse microscopy. Red dots mark the end of the migration tracks. Every track starts at x = 0 / y = 0. **(B)** Tracks of n = 30 randomly selected DCs for each condition from 3 independent experiments were quantified and the chemotactic index was determined with Chemotaxis and Migration tool V2.0 (Ibidi). **(C)** Similarly, the speed of migration was quantified. Statistical significance was assessed using ANOVA with Bonferroni post-test. **(D)** DCs infected with wt- or Δ*sseI-Salmonella* (MOI = 30; 30 min) were lysed at the indicated times and immunoblot analysis was performed with indicated antibodies (GαQE, p-Akt, Akt and actin). **(E-G)** Migration of DCs after ectopical expression of wild type (wt) SseI or SseI-C178A (C178A). Cells were transferred into a collagen matrix, a CCL19 gradient was applied, and directed migration (E) was determined by time-lapse microscopy (wt SseI, n = 43; SseI-C178A, n = 49). Cells were randomly selected from 3 independent experiments. **(F, G)** Quantification of the chemotactic index (F) and speed (G) of DCs. Statistical significance was assessed using unpaired, two-tailed Students t-test.

Recent studies from the Monack laboratory showed that SseI inhibits directed cell migration [10, 13]. Therefore, we were prompted to study the role of G proteins in the action of SseI in more detail. To this end, we investigated the migration of dendritic cells in a 3D collagen model. We infected bone-marrow derived mature dendritic cells (DCs) with wt- or Δ*sseI-Salmonella*, seeded the cells into collagen gels and applied a CCL19 chemokine gradient. Δ*sseI*-infected cells migrated with comparable speed and directness towards the chemokine compared to non-infected cells. In contrast, wt-infected DCs completely lost their ability to migrate in a directed manner, confirming previous results [10, 13] (Fig 5A–5C; S1 Video). As found for macrophages, time-resolved Western blot analysis revealed deamidation of Gα-subunits by wt-*Salmonella* in DCs (Fig 5D). Moreover, very similar as observed in macrophages, downstream signaling of the PI3K pathway was elevated in wt-*Salmonella*-infected DCs, exhibiting increase in Akt phosphorylation (Fig 5D).

To investigate whether the inhibition of migration observed with wt-*Salmonella* was due to the enzymatic activity of SseI, we ectopically expressed wt SseI or the inactive C178A-SseI mutant in DCs. As shown in Fig 5E and 5F (see also S2 Video), transfection with wt *sseI* led to a similar loss of chemotaxis as compared to infection with wt-*Salmonella*, while cells transfected with SseI-C178A mutant migrated normally. Interestingly, we did not detect a difference in the speed of migration between wt- and inactive *sseI*-transfected cells (Fig 5G).

### Persistent Gα_i_ activation inhibits directed migration of dendritic cells

**Fig 6:**
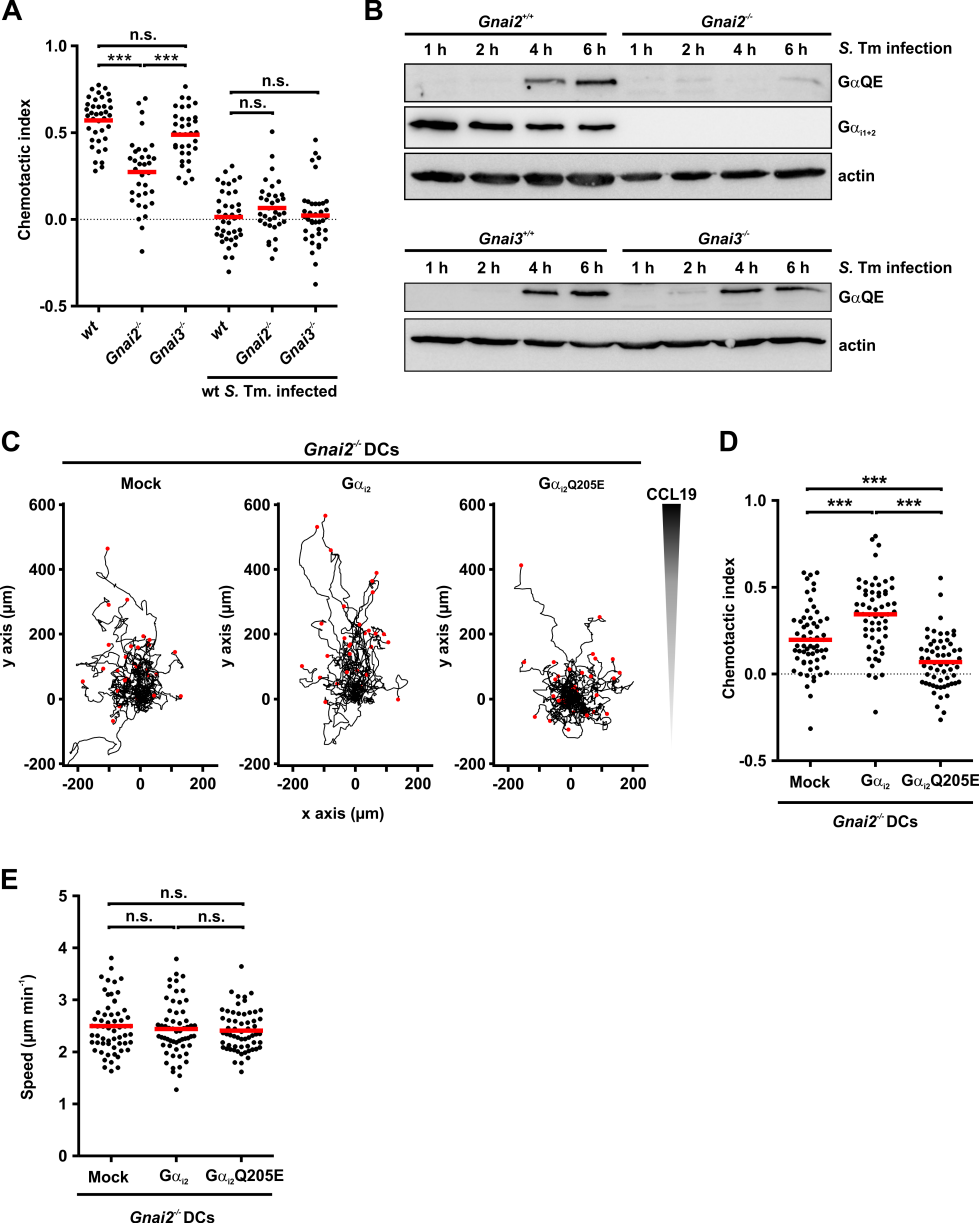
Effects of G protein deamidation on chemotaxis. **(A)** Wild type (wt)-, *Gnai2*^*-/-*^*—and Gnai3*^*-/-*^*—*DCs were infected with wt-*Salmonella* (wt *S*. Tm) (MOI = 30; 30 min) and the chemotactic indices were determined from at least n = 34 cells for each condition from 3 independent experiments as in Fig 5. Statistical significance was assessed using ANOVA and Bonferroni post-test. **(B)** Time-resolved immunoblot analysis of DCs derived from *Gnai2*^*-/—*^(upper panel), *Gnai3*^*-/—*^(lower panel) mice or wild type littermates (*Gnai2*^*+/+*^, upper panel; *Gnai3*^*+/+*^, lower panel) after infection with wild type *Salmonella* (S. Tm infection) (MOI = 30, 30 min) for indicated times. Proteins were analyzed by specific antibodies (GαQE, Gα_i1+2_ and actin). **(C-E)** *Gnai2*^*-/-*^ DCs were transfected with Gα_i2_, Gα_i2_Q205E or empty vector. Chemotaxis in a collagen matrix (C) with CCL19 gradient was measured by cell tracking using time-lapse microscopy. Chemotactic index (D) and migration speed (E) were calculated as described in Fig 5. Statistical significance was assessed using ANOVA (Bonferroni post-test).

To gain further evidence, whether deamidation of Gα_i_-subunits is the cause for impaired chemotaxis, we investigated the directed migration of DCs derived from *Gnai* deleted mice. The 3 Gα_i_ isoforms (Gα_i1,2,3_) are encoded by *Gnai1*, *2* and *3*. Relevant for DCs are Gα_i2,3_ (see Discussion section). Gα_i1_ is only poorly or not at all expressed in dendritic cells [37] (http://www.immgen.org/databrowser/index.html). We purified Gα_i2_ and Gα_i3_ as recombinant proteins and performed *in vitro* deamidation assays. Both isoforms of the Gα_i_-subunit are deamidated in a comparable manner (S4A Fig). Because double *Gnai2*^*-/-*^ / *Gnai3*^*-/-*^ mice are not viable [38], we obtained DCs from *Gnai2*^*-/-*^ or *Gnai3*^*-/-*^ C57BL/6 mice. In uninfected cells (controls), depletion of Gα_i2_ (*Gnai2*^*-/-*^) impaired chemotaxis as compared to wild type DCs, while Gα_i3_ depletion (*Gnai3*^*-/-*^) had no major effect on migration (Fig 6A). Thus, as reported for macrophages [39], our data indicated a crucial role of Gα_i2_ but not of Gα_i3_ in directed migration of DCs. Infection with wt-*Salmonella* impaired chemotaxis of control, Gα_i2_- and Gα_i3_-depleted DCs (Fig 6A), while migration speed was not significantly altered (S4B Fig). As expected, immunoblot analysis with anti-Gα_i1,2_ antibody revealed deletion of Gα_i2_ in *Gnai2*^*-/-*^ DCs (Fig 6B). While infection of control *Gnai2*^*+/+*^ cells with wt-*Salmonella* resulted in strong labeling of deamidated G proteins after 6 h, almost no labeling was observed in *Gnai2*^*-/-*^ DCs (Fig 6B). In *Gnai3*^*-/-*^ DCs deamidation was observed in a similar intensity as in *Gnai3*^*+/+*^ cells (Fig 6B). This indicates that Gα_i2_ is the predominant SseI substrate in mouse DCs. To mimic SseI-induced deamidation, we expressed mutant Gα_i2_Q205E or wt Gα_i2_ in Gnai2^-/-^ DCs. Expression was monitored by immunoblotting (S4C Fig). Ectopic expression of wt Gα_i2_ partially rescued the impaired chemotaxis of *Gnai2*^*-/-*^ DCs, while expression of mutant Gα_i2_Q205E strongly inhibited directed migration of DCs (Fig 6C and 6D, and S3 Video). Again, the migration speed was not changed by expression of Gα_i2,_ or Gα_i2_Q205E (Fig 6E).

## Discussion

Our studies elucidate the molecular mechanism of the *S*. Typhimurium SPI-2 T3SS effector SseI. This effector protein plays a crucial role in *S*. Typhimurium infection after invasion of the pathogen and modulates the immune responses of the host [9]. Here, we show that SseI deamidates a specific glutamine residue (Gln205 in Gα_i2_) in the α-subunits of heterotrimeric G proteins, which is involved in GTP hydrolysis, thereby the G protein is persistently activated. Thus, T3SS effector SseI exhibits the same mode of action as exotoxin PMT from *Pasteurella multocida*. This finding is in line with the structural similarity of both toxins, although the primary sequence identity of the catalytic domains is only ~ 20% [16]. Exchange of the conserved catalytic amino acid residues C178, H216 and D231 inhibited the deamidase activity of SseI. Because SseI is per se not able to enter target cells, we constructed a chimeric toxin (PMT-SseI^C^), consisting of the N-terminal binding and translocation domain of PMT and the deamidating domain of SseI. Additionally, we transfected mammalian cells with a GFP-SseI construct. These experiments confirmed our *in vitro* results in intact cells, showing that SseI acts as a deamidase on heterotrimeric G proteins.

While PMT deamidates several G proteins, including G_i/o_, G_q/11_ and G_12/13_ [23, 24], our data indicate that in intact cells, SseI is specific for G_i_ proteins. In line with activation of G_i_, SseI decreased cAMP levels in cells. Besides inhibition of adenylyl cyclase, G_i_ is crucially involved in activation of PI3 kinase via release of Gβγ subunits [29, 40]. Accordingly, we observed the translocation of a PIP_3_ sensor protein (GFP-Grp_PH_) to the cell membrane after treatment with PMT-SseI^C^. Many reports describe increase in Akt/mTOR signaling in *Salmonella*-host interactions [33, 36, 41]. We found that SseI increased phosphorylation and activation of Akt, its target mTOR and S6 ribosomal protein. These effects depended on the active SseI protein. The inactive mutant SseI-C178A had no effect.

To study the role of SseI in infection, we employed *S*. Typhimurium, in which SseI was deleted. These studies confirmed that after infection of macrophages with wt-*S*. Typhimurium, G_i_ proteins were deamidated, while this was not observed with Δ*sseI*-*Salmonella*. Importantly, deamidation of G proteins was detected only after uptake of *Salmonella* into host cells, indicating the importance of invasion for activation of SPI-2 T3SS and translocation of the effector SseI. wt-*Salmonella* strongly activated the pro-survival kinase Akt in macrophages and DCs. Notably, also infection with Δ*sseI*-*Salmonella* resulted in Akt phosphorylation. However, the phosphorylation of Akt, observed in the absence of SseI, diminished with time, whereas wt-*Salmonella* caused longer lasting Akt phosphorylation. Short-term Akt activation by Δ*sseI*-*Salmonella* is probably caused by *Salmonella* outer protein B (SopB), which is an effector of SPI-1 T3SS and a strong activator of Akt [42, 43]. Activation of Akt by SopB differs from SseI and might be independent of class I PI3 kinase [44, 45]. In contrast, we observed that activation of Akt by SseI is inhibited by the class I PI3K inhibitor LY29. In line with the activation of the PI3K-Akt signaling pathway, we observed increased survival of host cells after infection with wt-*Salmonella*, as compared to Δ*sseI-*infection.

Previously, it was reported that SseI inhibits the directed motility of macrophages and DCs towards a chemokine gradient [13]. Using an infection model, we confirmed that wt-*Salmonella* but not Δ*sseI*-*Salmonella* inhibited directed migration of DCs. Until recently, the molecular mechanism by which SseI affects directed migration was largely enigmatic. Our data indicate that the SseI-catalyzed deamidation of G_i_ proteins is responsible for this effect. Concomitantly with inhibition of directed migration, infection with wt-*Salmonella* but not with Δ*sseI*-*Salmonella* caused deamidation of G_i_ in DCs.

It is widely accepted that G_i_ proteins play a pivotal role in chemotaxis and most, if not all, chemokine receptors are coupled to G_i_ proteins [37, 46]. Three G_i_ proteins (G_i1-3_) exist, which share 85–95% amino acid sequence identity in their Gα subunits. While G_i1_ is only poorly expressed in leukocytes, G_i2_ and G_i3_ are highly expressed [37] (http://www.immgen.org/databrowser/index.html). Especially, G_i2_ appears to be essential for immune cell signaling. Several studies showed that G_i2_ plays an important role in directed migration and homing of B and T-cells [47–50] and in macrophage migration [39]. Similarly, we show that deletion of Gα_i2_ inhibits the migration of DCs towards a CCL19 gradient. In line with recent publications, deletion of Gα_i3_ had minor effects on directed DC migration [39, 51]. However, it is notable that wt-*Salmonella* infection was still able to inhibit directed migration even in *Gnai2*^-/-^ DCs, possibly indicating that Gα_i3_ is able to substitute in part for Gα_i2_. When wild type Gα_i2_ was re-expressed in *Gnai2*^-/-^ DCs, directed migration was partially rescued. Importantly, expression of the mutant Gα_i2_-Q205E that mimics the action of SseI, inhibited DC migration. Moreover, our studies reveal that SseI-induced deamidation of G_i_ proteins inhibits directed migration but does not affect the migration speed. Also pertussis toxin was shown to inhibit directed migration of DCs without affecting migration speed [52]. However, in contrast to SseI, pertussis toxin inactivates Gi proteins by blocking the interaction with GPCRs.

Thus, how might SseI, which activates Gi proteins, inhibit directed cell migration? McLaughlin and coworkers demonstrated that SseI binds to the scaffold protein IQGAP1 (Ras GTPase-activating-like protein) [13], which interacts with numerous proteins (e.g. Rho proteins) involved in regulation of actin dynamics [53]. Because IQGAP1 also binds to the inactive SseI mutant, this cannot explain the action of SseI [13]. Earlier studies, which were mainly based on two-hybrid screen analyses, found interactions of SseI with the actin-binding protein filamin and the LIM domain fragment of TRIP6 [54, 55]. However, these studies did not consider an enzyme activity of SseI. We propose that inhibition of directed migration by SseI is caused by blockade of the GTPase cycle and the non-polarized persistent activation of G_i_. SseI blocks rapid deactivation and activation of G_i_, which is probably essential to follow a chemoattractant gradient. In line with this hypothesis are recent findings about RGS (regulators of G protein signaling) proteins in immune cells. RGS proteins decrease the activity of G proteins by accelerating GTP hydrolysis and, thereby, accelerate G protein inactivation but also its activation [56–58], which appears to be essential for chemotaxis [8, 37]. It was shown that immune cells, harboring the Gα_i2_ G184S mutant, which causes resistance to RGS proteins, exhibit major alteration in chemotaxis [8]. The active state of the G184S Gα_i2_ mutant is prolonged, a condition mimicking the effects of SseI that persistently activates G_i_ proteins. Thus, a similar scenario as seen with the G184S Gα_i2_ mutant but even stronger, might be induced by SseI, resulting in insufficient downstream signaling from GPCRs-involved in chemotaxis and loss of polarization necessary for directed migration. In line with this hypothesis are also previous findings obtained with constitutively activated PI3Kγ, which was fused with the CAAX box of K-Ras resulting in persistent cell membrane localization and non-polarized enhancement of PIP_3_ production [59]. Leukocytes with constitutively activated PI3Kγ lost cell polarity and failed to follow a chemotactic gradient but they did not show differences in mean migration velocity. Surve and coworkers reported that selective activation of Gβγ by compound 12155 causes directed migration of neutrophils [60]. This effect was inhibited by Wortmannin, underlining the essential involvement of PI3K. PI3K is able to activate Rac, which is crucially involved in polarity control during neutrophil chemotaxis [61]. A recent study performed with optogenetic activation of PI3K sheds some light on the complexity of signaling pathways involved in directed migration downstream of PIP_3_ [62]. The authors showed that PI3K activates Rac mainly via the guanine nucleotide exchange factor P-Rex-1. However, Rac activation by chemoattractants (involving GPCRs and not only PI3K) depended on multiple Rac regulators indicating involvement of a signaling network.

Taken together our data elucidate the molecular mode of action of the SPI-2 effector SseI by deamidation of a crucial glutamine residue in the α-subunits of G_i_ proteins of the host, which results in inhibition of the turn-off mechanism of the G protein. The finding that G_i_ proteins are persistently activated by SseI will offer a new perspective in studies on the biological effects of this SPI-2 T3SS effector and may help to identify further signal factors involved in chemotaxis, acting downstream of G_i_ proteins.

## Materials and methods

### Materials

PCR primers were from Apara (Denzlingen, Germany). All other reagents were of analytical grade and purchased from commercial sources. All Antibodies used in this study are listed in S1 Table.

### Bacterial strains

*S*. *enterica* serovar Typhimurium NCTC12023 (ATCC 14028) was used as parent strain for the Δ*sseI* strain, which was created by deletion of the SseI coding sequence [63].

### Mouse primary cell cultures

Murine BMDMs and DCs were isolated and cultivated as described before [64, 65] with adjustments. 6–12 weeks old wt (Charles River), *Gnai2*^*-/-*^, and *Gnai3*^*-/-*^ (Dr. B. Nürnberg, maintained at animal facility, Medizinische Fakultät und ICePhA, University of Tübingen) mice (C57BL/6N, female, [66]) were euthanized and femurs and tibiae were extracted. Bones were cut open and bone marrows were flushed with sterile, ice-cold PBS. Cells and matrix were resuspended and pipetted through a 70 μm cell strainer. Cell suspension was centrifuged (300 x g, 5 min). Supernatants were discarded, pellets were resuspended in RPMI-1640 with 10% fetal calf serum (FCS) and 1% penicillin/streptomycin (P/S).

Dendritic cells: Cell concentration was adjusted to 2.5 x 10^5^ cells/ml and 10 ml suspension was added into 10 cm bacterial petri dishes. GM-CSF was added for a final concentration of 20 ng/ml. At day 3, 10 ml medium with 20 ng/ml GM-CSF was added. At day 6, 10 ml medium were carefully removed and 10 ml fresh medium with 20 ng/ml GM-CSF were added. At day 8–9, non-adherent cells were collected and centrifuged (300 x g, 5 min). Pellets were resuspended in 10 ml fresh medium with 20 ng/ml GM-CSF without P/S and transferred into 6 cm cell culture dishes. LPS (200 ng/ml) were added for 24 h for further maturation of the DCs, when needed. Only non-adherent cells were used for experiments.

Bone marrow-derived macrophages: Cell concentration was adjusted to 5–7 x 10^5^ cells/ml and 10 ml suspension was added into 10 cm tissue culture dishes. M-CSF was added for a final concentration of 20 ng/ml. At day 2, 10 ml fresh medium with 20 ng/ml M-CSF were added. At day 4, 10 ml medium were removed and exchanged by 10 ml fresh medium with 20 ng/ml M-CSF. At day 6–8, medium and non-adherent cells were discarded, adherent cells were detached with trypsin/EDTA and plated in medium with 20 ng/ml M-CSF without P/S at desired concentrations for experiments.

### Cell lines

HEK-293 (female; ACC 305 from DSMZ, Germany) and HeLa (female, ATCC CCL-2) cells were cultured in Dulbecco's modified Eagle's medium (DMEM) supplemented with 10% FCS and 1% P/S in an atmosphere of 5% CO_2_ at 37°C. RAW264.7 cells (male, ATCC TIB-71) were cultured in DMEM supplemented with 5% FCS and 1% P/S.

### Plasmids

SseI encoding DNA was amplified from *S*. *enterica* serovar Typhimurium NCTC12023 and cloned into the bacterial expression vector pCOLDII and mammalian expression vectors pEGFP-C1 and pcDNA3.1 by standard cloning techniques. Additionally, a C-terminal fragment of SseI harboring the homology domain to PMT-C3, starting with amino acid 137 was cloned into pCOLDII. Chimeric PMT-SseI^C^ consists of the N-terminal portion (amino acid 1–505) of PMT and the PMT-C3 homology domain of SseI (amino acids 137–322). PMT-SseI^C^ was cloned in accordance to the method described before for other PMT fusion proteins into the pGEX2T vector [26]. The biological inactive mutant of SseI (C178A) was constructed by site-directed mutagenesis. Gα_i2_ DNA (with optimized codon usage for prokaryotic expression) was ordered as gBlock (Integrated DNA Technology, IDT) and cloned into pCOLDII. All oligonucleotides used in this study are listed in S2 Table.

### Transfection, infection

HeLa cells were transfected using PEI as described previously [67]. Transfection of RAW264.7 cells was performed utilizing lipofectamin2000 (Thermo Fisher Scientific) according to the manufacturer’s instructions. Transfection of BMDMs and BMDCs was done using amaxa nucleofector kits (Lonza) according to manufacturer’s instructions. For *in vitro* infection studies *S*. *enterica* serovar Typhimurium NCTC12023 and the isogenic variant MvP393Δ*sseI* were used. BMDMs or RAW 264.7 cells were infected with *Salmonella* with indicated MOIs for 30 min. Then cells were washed 3 times with warm PBS and incubated with DMEM (RAW264.7) or RPMI-1640 (BMDMs) supplemented with 100 μg/ml gentamicin for 60 min. For the remaining time of the experiment, the medium was changed to DMEM or RPMI supplemented with 10 μg/ml gentamicin. For immunofluorescence detection of deamidation in RAW264.7 macrophages, these cells were infected with *Salmonella* for 5 h (MOI = 1). Dendritic cells were infected with a MOI = 30 for 30 min. Due to non-adherence of stimulated DCs, cells were resuspended in infection-medium and centrifuged (300 x g / 5 min). Pellets were resuspended in RPMI-1640 containing 100 μg/ml gentamicin and further treatment of the cells was similar to treatment of BMDMs.

For lysates cells were treated with RIPA buffer (1 mM EDTA, 25 mM Tris, 150 mM NaCl, 1% (v/v) Triton X-100, and 1% (m/v) sodium deoxycholate, pH 7.4), containing complete protease inhibitor (Roche) and phosphatase inhibitor (phosphatase inhibitor cocktail 2/3, Sigma) for 20 min on ice with occasional vortexing. Lysates were then centrifuged at 4°C (10,000 rpm for 10 min).

### FRET measurements

cAMP was measured by fluorescence resonance energy transfer (FRET) using a single chain cAMP sensor as described before [27]. The construct EPAC2-camps was kindly provided by Dr. Viacheslav O. Nikolaev (University of Göttingen, Germany).

### cAMP measurements

HEK-293 cells were incubated with wildtype PMT-SseI or inactive mutant (PMT-SseI-C178A) for 8 h. Then forskolin (10 μM) and 3-isobutyl-1-methylxanthin (IBMX 100 μM) were added to the medium to induce adenylyl-cyclase activity and cells were incubated for further 45 min. Medium was discarded and cells were lysed with lysis buffer from the cAMP Parameter Kit (Biotechne). Further treatment of the samples was performed according to the manufacturer’s instructions (cAMP Parameter Kit/R&D Systems Biotechne).

### Confocal Laser Scanning Microscopy of GFP-Grp1_PH_ translocation

The subcellular distribution of GFP-Grp1_PH_ reflects the activity of PI3Ks, i.e. enhancement of membrane-associated fluorescence was taken as an indicator of PI3K activation. Assay was performed as previously described [31].

### Fluorescence microscopy

For fluorescence microscopy, cells were prepared as described before [26]. Fixed samples were analyzed with an inverted Axiovert 200 M microscope (Carl Zeiss) with a Yokogawa (Tokyo, Japan) CSU-X1 spinning disc equipped with an emission filter wheel and 405 nm / 488 nm / 561 nm laser lines.

### Protein expression

SseI and Gα_i2_(His) were expressed as N-terminal His_6_-tagged proteins and purified by affinity chromatography via a Ni-NTA column. Expressions of PMT or chimeric constructs of PMT and SseI were performed as glutathione-S-transferase fusion proteins in accordance with the method previously described [67]. Coexpression of SseI or the inactive mutant (SseI-C178A) with GST-Gα_i2_ was done as previously described [23]. G proteins used for the *in vitro* deamidation assay in S4A Fig were purified as GST-fusion proteins. The GST-tag was cleaved with Thrombin.

### *In vitro* deamidation assay

Purified Gα_i2_ or Gα_i3_ (1 μM) was preincubated in deamidase buffer (50 mM sodium HEPES (pH 7.5), 50 mM NaCl, 1 mM EDTA, 5 mM MgCl_2_ and 0.1% NP-40) together with Guanosine diphosphate (GDP, 5 μM) for 15 min at 30°C. SseI-C deamidase domain (100 nM) was added and reaction was performed at 16°C for 16 h. Reaction was stopped by the addition of 5x Lämmli buffer and shock freezing in liquid N_2_, followed by immunoblot analysis using the deamidation specific GαQE antibody.

### Immunoblot analysis

For immunoblotting, samples were subjected to SDS-PAGE and transferred onto polyvinylidene difluoride-membrane. Deamidation specific antibody anti-Gα_q_ Q209E (3G3) was used as described before [25]. Binding of the appropriate horseradish peroxidase-coupled secondary antibody was detected by using enhanced chemiluminescent detection reagent (New England Biolabs) and the imaging system LAS-3000 (Fujifilm).

### Mass spectrometric analysis

Gel bands were destained for 10 min using 30% acetonitrile / 70% 0.1 M NH_4_HCO_3_, followed by 10 min washing with 0.1 M NH_4_HCO_3_; this procedure was repeated twice. Prior to drying in a Vacuum Concentrator (Concentrator 5301, Eppendorf), gel bands were shrunk with 100% acetonitrile. By applying 0.1 μg trypsin per gel band, proteolytic digest was performed overnight at 37°C in 0.1 M NH_4_HCO_3_. Peptides were extracted from the gel matrix with 5% formic acid. Peptides were analyzed by LC/MS on a Q-TOF mass spectrometer (Agilent 6520, Agilent Technologies) coupled to a 1200 Agilent nanoflow system via a HPLC-Chip cube electrospray ionization interface carrying a ProtID-II-Chip (Agilent Technologies). For peptide separation, a linear gradient ranging from 3% acetonitrile (ACN) to 30% ACN running for 30 min at a flow rate of 300 nl/min was used. MS spectra were acquired from *m/z* 50 to *m/z* 3000 while operating the Q-TOF in the 2 GHz extended dynamic range mode. Internal mass calibration was enabled. Three multiple charged peptides were selected in a data-dependent manner from each survey MS-scan for acquisition of fragment mass spectra. For peak list generation, raw data files were processed using Mascot Daemon2.4.0 with default settings for the Agilent Q-TOF. Using Mascot Server 2.4.1 database searches were performed with tryptic specificity and two missed cleavages against a small database comprised of known contaminants and the target sequence. Mass tolerances of 50 ppm and 0.05 Da were allowed for peptide and fragment masses, respectively. As variable modifications deamidation of glutamine and phosphorylation of serine and threonine were selected in addition to Carbamidomethyl (C), Gln-> pyroGlu (N-term. Q) and oxidation (M). Extracted Ion Chromatograms were generated using the Qualitative Analysis module within the MassHunter B04.00 software.

### LDH-release/cell viability

RAW264.7 macrophages were infected with a MOI of 30. 1.5 h p.i. medium was exchanged by Earle’s balanced salt solution (EBSS) containing 10 μg/ml gentamicin and LY29 (30 μM), where indicated. Further treatment of the cells was performed according to the manufacturer’s instructions (Promega CytoTox-ONE/CellTiter-Blue). In-plate fluorescence was acquired with the Tecan Infinite M200 plate reader.

### 3D dendritic cell migration

Chemotactic migration assays of dendritic cells were performed as described before [64]. Briefly, pre-treated dendritic cells were mixed with bovine collagen (1.5 mg/ml) and incubated in self-built migration chambers at 37°C, 5% CO_2_ until the collagen network was polymerized. The collagen-cell mixture was covered with medium, containing CCL19 (500 ng/ml), which diffuses into the gel matrix and thus forming the chemokine gradient. Cell migration was monitored with a Lionheart FX automated microscope. Cell tracks were analyzed with ImageJ and the Manual Tracking plugin, followed by evaluation of the data with Ibidi chemotaxis and migration tool. Calculation of the chemotactic indices:

$$Chemotactic\;index=\frac{1}{n}\sum_{i=1}^{n}\frac{y_{i\;(distance\;migrated\;parallel\;to\;chemokine\;gradient)}}{d_{i\;(accumulated\;migration\;distance)}}$$

### Statistics

Results are presented as means ±SEM, unless otherwise stated. Statistical analyses were performed using GraphPad PRISM Version 5.04. Significance was assessed by Student`s t-test and Mann-Whitney test, depending on whether the data was normally distributed. p values < 0.05 were considered statistically significant (* = p < 0.05; ** = p < 0.01;*** = p < 0,001; ns, not significant). Multiple group comparisons were analyzed by ANOVA and Bonferroni post-tests.

### Ethics statement

All animal experiments were performed in compliance with the German animal protection law (TierSchG). The animals were housed and handled in accordance with good animal practice as defined by FELASA (www.felasa.eu) and the national animal welfare body GV-SOLAS (www.gv-solas.de). The animal welfare committees of the University of Freiburg as well as the local authorities (Regierungspräsidium Freiburg, licenses X-13/03A and X-17/01F) approved all animal experiments.

## Supporting information

S1 Fig Identification of SseI as a deamidase homologous to PMT. **(A)** Alignment of the deamidase domain of PMT with SseI (UniProt accession no.: PMT, P17452; SseI, Q8ZQ79). Alignment was performed with ClustalO (Sievers et al., 2011). Catalytic triad of PMT deamidase domain is highlighted by blue boxes. **(B, C)** Electron-transfer dissociation (ETD) tandem MS/MS spectrum of (B) m/z 455.2170^(2+)^ and of (C) m/z 455.7102^(2+)^ showing a shift of 1 Dalton from y2 upwards, indicating a deamidation of glutamine-205 to glutamic acid.
(TIF)

S2 Fig Activation of PI3K pathways by SseI. **(A)** PMT-SseI^C^ blocks forskolin-induced cAMP accumulation. HEK-293 cells were incubated with 100 nM PMT-SseI^C^ or the inactive mutant (C178A) for 8 h. Cells were then incubated with forskolin (10 μM) and IBMX (100 μM) for 45 min. Cells were lysed and cAMP levels determined by cAMP Parameter Assay (Biotechne). Shown are data as means ±SEM from 4 independent experiments. Significance was assessed by Student`s *t*-test. **(B)** PI3Kγ activation by PMT-SseI^C^. All HEK-293 cells were transfected with the PI3K subunit p110γ, transfection with the PI3K subunit p101 was as indicated. Shown are confocal images of representative cells before (-fMLP) and 4 min after stimulation with fMLP (+fMLP). (panel a) Transfection without p101. (panels b-d) transfection with p101. Incubation of cells with PMT-SseI^C^ (panel c) or the inactive C178A mutant of PMT-SseI^C^ (PMT-SseI^C^C178A) (panel d, each 100 nM). The lower panels show the fluorescence line scan through cells representing the membrane translocation of the PIP_3_ sensor GFP-Grp1_PH_ upon treatment without and with PMT-SseI^C^, inactive PMT-SseI^C^C178A and fMLP (gray curve, before fMLP; black curve, after fMLP). **(C)** Densitometric quantification and statistical analysis of n = 3 immunoblots from cell lysates treated as described in Fig 2F. Statistical significance was assessed using one sample *t*-test, with *p<0.05, **p<0.01. **(D)** Influence of GFP-SseI transfection of RAW264.7 cells on phosphorylation of Akt (p-Akt) and S6 ribosomal protein (p-S6). p-Akt, p-S6 and actin were detected by specific antibodies after transfection of cells with wt GFP-SseI or inactive mutant GFP-SseI-C178A. **(E)** Quantification of the amount of p-S6 from S2D Fig. Phosphorylation of p-S6 after transfection with GFP-SseI was set 100, phosphorylation after GFP-SseI-C178A transfection is given as percent of maximum. Shown are means ±SEM of 3 independent experiments.
(TIF)

S3 Fig Deamidation of G Proteins in the infection model of *S*. Typhimurium. **(A)** Fluorescence microscopy of fixed BMDMs infected with wild type (wt-) or *ΔsseI-S*. Typhimurium. Macrophages were infected for 60 min with a MOI of 15 and washed. Cells were fixed and stained for deamidation (GαQE—green) and *Salmonella* O antigen (red) 5 h p.i.. Orthogonal views, cutting the z-stacks, show intracellular localization of the *Salmonella*. **(B)** Quantification of fluorescence intensity of deamidation of n = 30 cells without and with infection by wild type (wt)- or *ΔsseI-S*. Typhimurium from 3 independent experiments. Cells were treated as described in (A). Gα protein deamidation of *Salmonella*-infected or uninfected cells was calculated by determining the average intensity of Alexa 568 fluorescence of the whole cell area in the Z plane with internalized Salmonella. Means ±SD are shown. Significance was assessed by ANOVA. **(C)** Quantification of p-Akt immunofluorescence. RAW264.7 cells were infected with wt- or *ΔsseI-S*. Typhimurium or remained non-infected as shown in Fig 3C. Fluorescence intensity of n = 45 cells for each condition from three independent experiments was measured. Statistical significance was assessed using ANOVA.
(TIF)

S4 Fig Effects of *Salmonella* infection on migration speed of *Gnai*-deleted DCs and immunoblot analysis of Gα_i2_ expression of *Gnai2*^*-/-*^ in DCs. **(A)** *In vitro* deamidation of G protein isoforms Gα_i2_ and Gα_i3_. Immunoblot analysis of the recombinantly expressed G proteins incubated with wild type C-terminal part of SseI^C^ (wt) or mutant SseI^C^ (C178A). **(B)** Quantification of the migratory speed of DCs obtained from wild type (wt)-, *Gnai2*^*-/-*^ or *Gnai3*^*-/-*^ mice. Cells were infected with wild type (wt S. Tm.) *S*. Typhimurium (MOI = 30, 30 min) and, thereafter, transferred into migration chambers. Infected and non-infected DCs from each condition (n≥34 from 3 independent experiments) were tracked and migratory speed was calculated. **(C)** Immunoblot analysis of transfected cells with indicated antibodies (GαQE, Gα_i1+2_ and actin). DCs were transfected with pcDNA constructs, encoding for wild type Gα_i2_ or mutant Gα_i2_Q205E. Mock indicates empty vector transfection. Lysates were prepared 16 h post transfection. Representative blots from n = 3 experiments are shown.
Statistical significance was assessed using ANOVA.
(TIF)

S1 Video Time-lapse video (4 h) of DCs migrating after infection with GFP-expressing wt- or Δ*sseI-Salmonella* in a CCL19 gradient. Arrows indicate 2 examples of infected migrating cells.
(AVI)

S2 Video Time-lapse video (4 h) of DCs ectopically expressing wt SseI (left) or mutant SseI-C178A (right) in a CCL19 gradient. Tracks of migrating cells are shown.
(AVI)

S3 Video Time-lapse video (4 h) of Gnai2-/- DCs ectopically expressing wt Gα_i2_ or mutant Gα_i2_Q205E in a CCL19 gradient. Tracks of migrating cells are shown.
(AVI)

S1 Table Antibodies used in this study. (XLSX)

S2 Table Oligonucleotides used in this study. (XLSX)
