# Supplementary figures and images for: Salmonella Typhimurium effector SseI inhibits chemotaxis and increases host cell survival by deamidation of heterotrimeric Gi proteins
Source: PLoS Pathog. 2018 Aug 13;14(8):e1007248. doi: 10.1371/journal.ppat.1007248 (PMC6107295; doi:10.1371/journal.ppat.1007248)

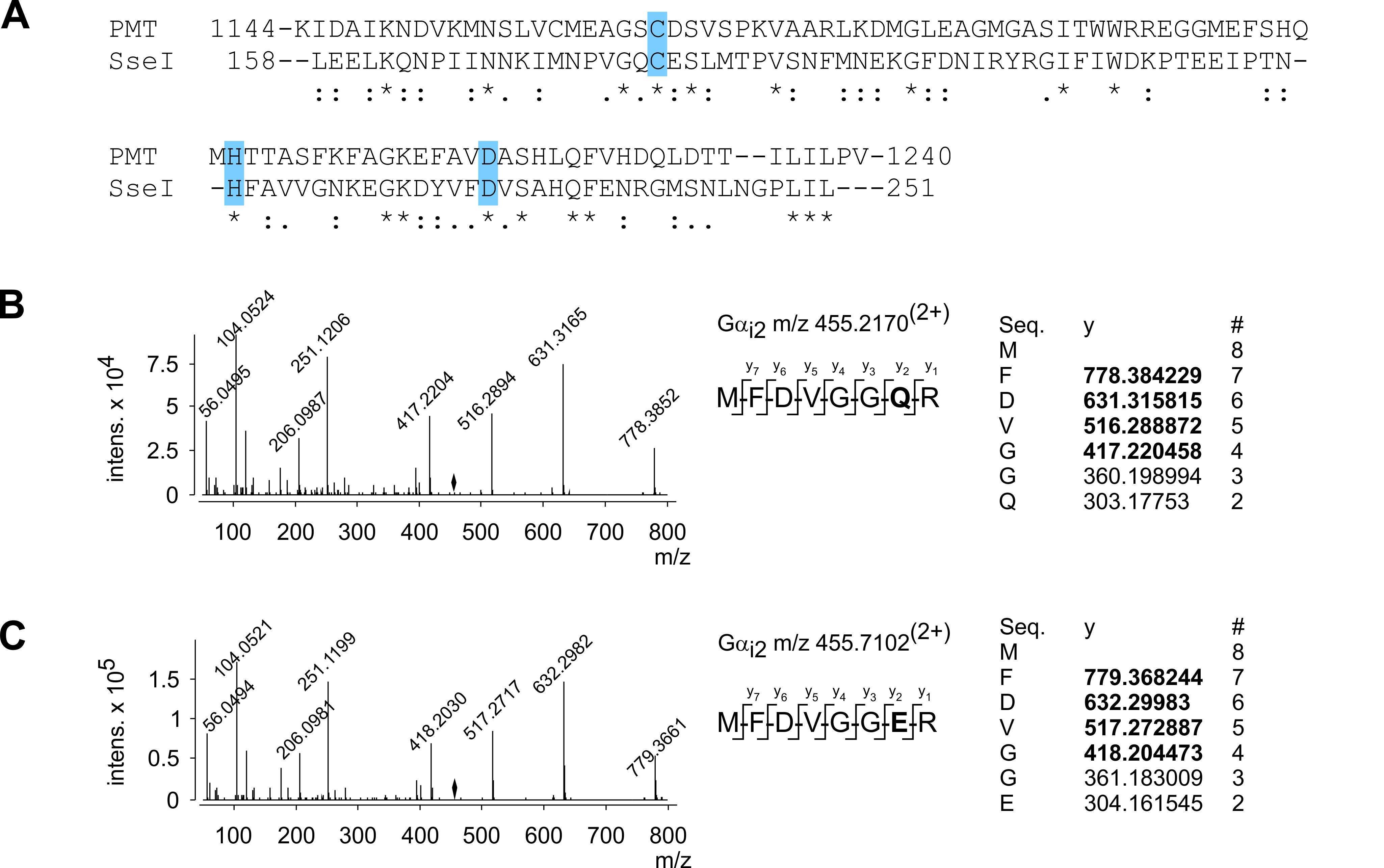

Supplement: S1 Fig — (A) Alignment of the deamidase domain of PMT with SseI (UniProt accession no.: PMT, P17452; SseI, Q8ZQ79). Alignment was performed with ClustalO (Sievers et al., 2011). Catalytic triad of PMT deamidase domain is highlighted by blue boxes. (B, C) Electron-transfer dissociation (ETD) tandem MS/MS spectrum of (B) m/z 455.2170(2+) and of (C) m/z 455.7102(2+) showing a shift of 1 Dalton from y2 upwards, indicating a deamidation of glutamine-205 to glutamic acid. (TIF) [file ppat.1007248.s001.tif]

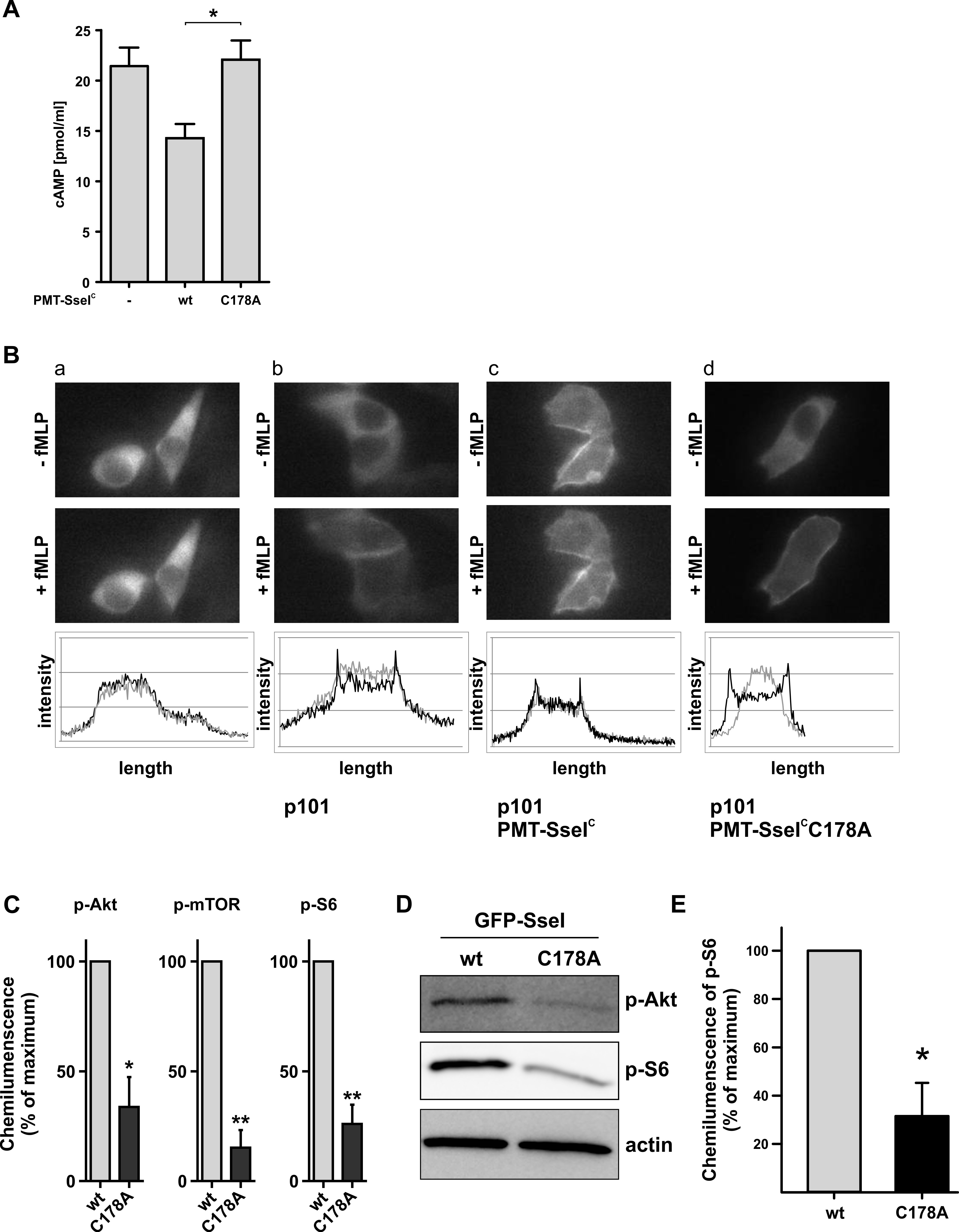

Supplement: S2 Fig — (A) PMT-SseIC blocks forskolin-induced cAMP accumulation. HEK-293 cells were incubated with 100 nM PMT-SseIC or the inactive mutant (C178A) for 8 h. Cells were then incubated with forskolin (10 μM) and IBMX (100 μM) for 45 min. Cells were lysed and cAMP levels determined by cAMP Parameter Assay (Biotechne). Shown are data as means ±SEM from 4 independent experiments. Significance was assessed by Student`s t-test. (B) PI3Kγ activation by PMT-SseIC. All HEK-293 cells were transfected with the PI3K subunit p110γ, transfection with the PI3K subunit p101 was as indicated. Shown are confocal images of representative cells before (-fMLP) and 4 min after stimulation with fMLP (+fMLP). (panel a) Transfection without p101. (panels b-d) transfection with p101. Incubation of cells with PMT-SseIC (panel c) or the inactive C178A mutant of PMT-SseIC (PMT-SseICC178A) (panel d, each 100 nM). The lower panels show the fluorescence line scan through cells representing the membrane translocation of the PIP3 sensor GFP-Grp1PH upon treatment without and with PMT-SseIC, inactive PMT-SseICC178A and fMLP (gray curve, before fMLP; black curve, after fMLP). (C) Densitometric quantification and statistical analysis of n = 3 immunoblots from cell lysates treated as described in Fig 2F. Statistical significance was assessed using one sample t-test, with *p<0.05, **p<0.01. (D) Influence of GFP-SseI transfection of RAW264.7 cells on phosphorylation of Akt (p-Akt) and S6 ribosomal protein (p-S6). p-Akt, p-S6 and actin were detected by specific antibodies after transfection of cells with wt GFP-SseI or inactive mutant GFP-SseI-C178A. (E) Quantification of the amount of p-S6 from S2D Fig. Phosphorylation of p-S6 after transfection with GFP-SseI was set 100, phosphorylation after GFP-SseI-C178A transfection is given as percent of maximum. Shown are means ±SEM of 3 independent experiments. (TIF) [file ppat.1007248.s002.tif]

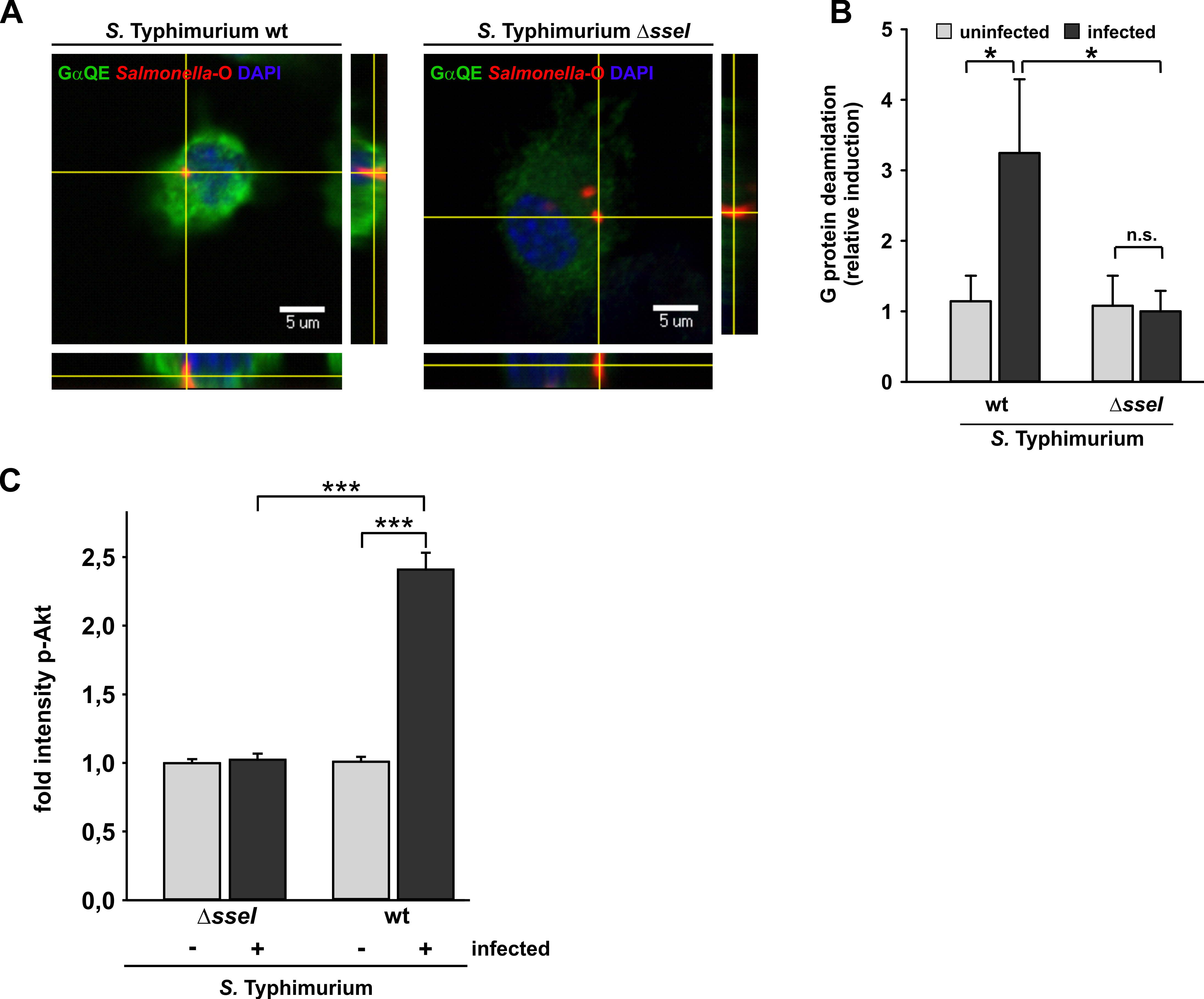

Supplement: S3 Fig — (A) Fluorescence microscopy of fixed BMDMs infected with wild type (wt-) or ΔsseI-S. Typhimurium. Macrophages were infected for 60 min with a MOI of 15 and washed. Cells were fixed and stained for deamidation (GαQE—green) and Salmonella O antigen (red) 5 h p.i.. Orthogonal views, cutting the z-stacks, show intracellular localization of the Salmonella. (B) Quantification of fluorescence intensity of deamidation of n = 30 cells without and with infection by wild type (wt)- or ΔsseI-S. Typhimurium from 3 independent experiments. Cells were treated as described in (A). Gα protein deamidation of Salmonella-infected or uninfected cells was calculated by determining the average intensity of Alexa 568 fluorescence of the whole cell area in the Z plane with internalized Salmonella. Means ±SD are shown. Significance was assessed by ANOVA. (C) Quantification of p-Akt immunofluorescence. RAW264.7 cells were infected with wt- or ΔsseI-S. Typhimurium or remained non-infected as shown in Fig 3C. Fluorescence intensity of n = 45 cells for each condition from three independent experiments was measured. Statistical significance was assessed using ANOVA. (TIF) [file ppat.1007248.s003.tif]

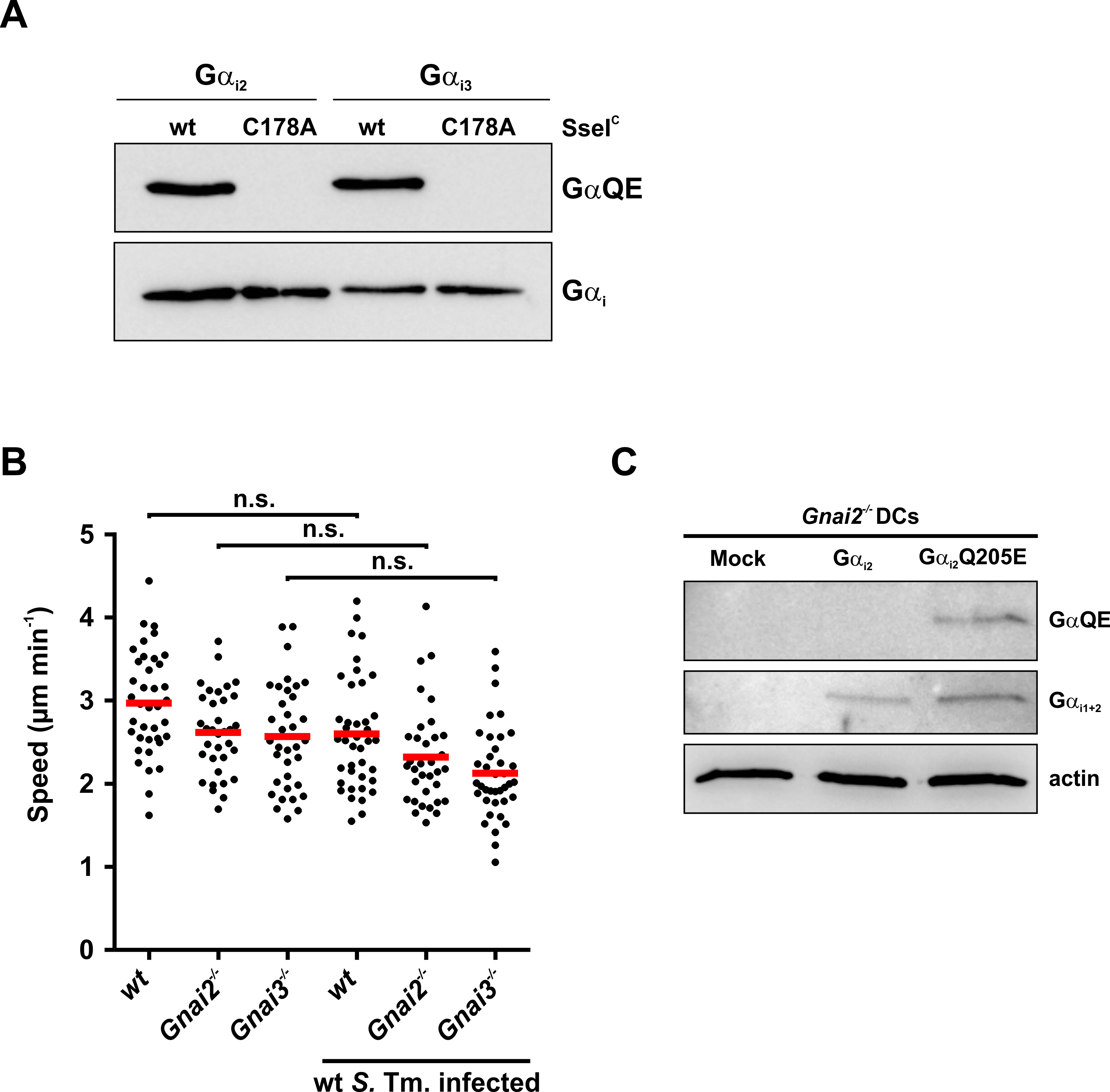

Supplement: S4 Fig — (A) In vitro deamidation of G protein isoforms Gαi2 and Gαi3. Immunoblot analysis of the recombinantly expressed G proteins incubated with wild type C-terminal part of SseIC (wt) or mutant SseIC (C178A). (B) Quantification of the migratory speed of DCs obtained from wild type (wt)-, Gnai2-/- or Gnai3-/- mice. Cells were infected with wild type (wt S. Tm.) S. Typhimurium (MOI = 30, 30 min) and, thereafter, transferred into migration chambers. Infected and non-infected DCs from each condition (n≥34 from 3 independent experiments) were tracked and migratory speed was calculated. (C) Immunoblot analysis of transfected cells with indicated antibodies (GαQE, Gαi1+2 and actin). DCs were transfected with pcDNA constructs, encoding for wild type Gαi2 or mutant Gαi2Q205E. Mock indicates empty vector transfection. Lysates were prepared 16 h post transfection. Representative blots from n = 3 experiments are shown. Statistical significance was assessed using ANOVA. (TIF) [file ppat.1007248.s004.tif]
